# The Role of Host Traits, Season and Group Size on Parasite Burdens in a Cooperative Mammal

**DOI:** 10.1371/journal.pone.0027003

**Published:** 2011-11-01

**Authors:** Hermien Viljoen, Nigel C. Bennett, Edward A. Ueckermann, Heike Lutermann

**Affiliations:** 1 Mammal Research Institute, Department of Zoology and Entomology, University of Pretoria, Pretoria, South Africa; 2 ARC-Plant Protection Research Institute, Queenswood, Pretoria, South Africa; 3 School of Environmental Sciences and Development, Potchefstroom University, Potchefstroom, South Africa; Biomedical Research Institute, United States of America

## Abstract

The distribution of parasites among hosts is often characterised by a high degree of heterogeneity with a small number of hosts harbouring the majority of parasites. Such patterns of aggregation have been linked to variation in host exposure and susceptibility as well as parasite traits and environmental factors. Host exposure and susceptibility may differ with sexes, reproductive effort and group size. Furthermore, environmental factors may affect both the host and parasite directly and contribute to temporal heterogeneities in parasite loads. We investigated the contributions of host and parasite traits as well as season on parasite loads in highveld mole-rats (*Cryptomys hottentotus pretoriae*). This cooperative breeder exhibits a reproductive division of labour and animals live in colonies of varying sizes that procreate seasonally. Mole-rats were parasitised by lice, mites, cestodes and nematodes with mites (*Androlaelaps* sp.) and cestodes (*Mathevotaenia* sp.) being the dominant ecto- and endoparasites, respectively. Sex and reproductive status contributed little to the observed parasite prevalence and abundances possibly as a result of the shared burrow system. Clear seasonal patterns of parasite prevalence and abundance emerged with peaks in summer for mites and in winter for cestodes. Group size correlated negatively with mite abundance while it had no effect on cestode burdens and group membership affected infestation with both parasites. We propose that the mode of transmission as well as social factors constrain parasite propagation generating parasite patterns deviating from those commonly predicted.

## Introduction

Parasites are an essential component of a healthy ecosystem and today it is widely recognised that they play a major role in shaping the community and population structure of hosts [1], [2], [3]. The distribution of parasites among a host population is often characterised by heterogeneity with a small number of hosts harbouring the majority of parasites [4], [5]. Such heterogeneities are caused by host as well as parasite-related properties and environmental factors that affect host exposure and susceptibility to parasites [4], [5]. Host-related factors include the sex of a host and indeed parasite loads often show a sex-biased pattern with males harbouring larger parasite loads than females [6], [7], [8]. Several alternative hypotheses have been proposed to explain such sex-biases in parasite load. Firstly, larger hosts may be able to sustain greater parasite populations as they represent larger resources. Since in most mammal species males represent the larger sex they consequently have higher parasite loads [7], [9]. Alternatively, the differential physiological make-up of the sexes (i.e. testosterone and oestrogen) may lead to sex differences in susceptibility to parasites and the assumed immunosuppressive properties of testosterone have frequently been invoked as a factor contributing to higher parasite loads in male vertebrates [8], [10], [11]. Lastly, behavioural differences between the sexes such as larger roaming ranges in males may contribute to differences in parasitism.

Other host-specific factors that affect parasite loads may be linked to the life-history trade-offs that a host experiences due to the limited amount of resources such as the energy available to an individual [12]. This has been particularly well studied for the trade-off between reproductive effort and parasite defence. Increases in parasite load during gestation, delays in the onset of breeding as well decreased reproductive success as a result of parasite burdens have been reported for females in a number of species e.g. [13], [14], [15], [16]. Similar trade-offs between reproductive effort (measured either as investment into sexually selected traits or parental effort) and parasite loads have been observed for males e.g. [17], [18]. These studies suggest that investment in reproduction results in higher susceptibility to parasites or that parasites divert resources away from reproduction.

The seasonality of reproduction in many organisms is thought to contribute to the commonly observed seasonal cycles in parasite loads among hosts [19], [20], [21]. The diversion of resources into reproduction as well as the recruitment of naive hosts could facilitate the successful propagation of parasites e.g. [22], [23]. Indeed some parasites appear to synchronise their reproduction with that of their hosts and peak loads are observed during the breeding season e.g. [13], [24]. Other parasites may be more prevalent during months when temperatures are lower and food is scarcer, since reduced energy availability can also compromise the host's immune system [19], [25]. In addition, seasonal changes in aggregation patterns of hosts such as breeding aggregations or the formation of over-wintering groups for thermoregulatory reasons e.g. [26], [27] can facilitate parasite transmission and result in seasonal fluctuations of parasite loads. Apart from these factors, parasites respond to external environmental cues such as temperature and humidity to proliferate, thereby affecting their seasonal pattern of abundance e.g. [28], [29], [30]. From the above it is apparent that seasonal patterns of parasite abundance can result from a number of factors that are not always mutually exclusive and their individual contributions may be difficult to disentangle.

Alexander [31] first suggested that parasites are a cost of sociality due to the frequency-dependent nature of parasite transmission. Indeed, a number of studies have found support for this hypothesis and parasite burdens increased with group size [2], [32], [33], [34]. However, other studies failed to find a correlation between group size and parasite load e.g. [35], [36], [37]. Wilson et al. [38] suggested that equating group size with host density and thus increased transmission rates may be overly simplistic. In contrast, reductions in inter-group transmission rates as a result of social structure may compensate for increases in intra-group parasite transmission. This hypothesis was further explored by Bordes et al. [39] who took group size as well as the degree of sociality into account. They found differential patterns of parasite burdens with sociality depending on the transmission mode of the parasite. Ectoparasite loads decreased significantly with increases in sociality while no correlation could be found for endoparasites across 46 rodent species. Bordes et al. [39] suggested that reductions in ectoparasite loads may be linked to increased grooming frequencies in larger groups or more social species while the dependence on intermediate hosts makes such simple correlations less likely for endoparasites. In another study on cooperatively breeding Galapagos falcons (*Buteo galapagoensis*) [34] found that the relationship between group size and louse infestation depended on the transmission mode of the parasite species. This was the first study of parasitism and sociality in a cooperative breeder but it focused exclusively on males during the breeding season.

The costs and benefits of cooperative breeding vertebrates, where individuals help to raise offspring other than their own, have been the focus of a large number of studies [40], [41], [42] but parasites have largely been neglected. The aim of the current study was to assess the contributions that host sex and reproductive status, season and sociality have on parasite burdens in the cooperatively breeding highveld mole-rat (*Cryptomys hottentotus pretoriae*). Highveld mole-rats live in groups of up to 14 individuals and exhibit a reproductive division of labour with only one female and up to three putative males engaging in procreation while the remaining group members are reproductively quiescent [43]. Non-breeding individuals of both sexes have reduced sexual hormone concentrations in comparison with their breeding counterparts and births are restricted to spring and early summer [44]. The animals regularly exhibit allogrooming which could reduce ectoparasite burdens in a group [45]. Mole-rats are subterranean rodents and as a result of the great energetic costs of digging [46] their movements are largely restricted by rainfall [47]. The extension of burrow systems occurs mostly during the wet season of the year e.g. [48] and is associated with dramatic increases in energy expenditure [49], [50]. Although the burrow pattern of social mole-rats is constantly changing, the overall home range appears to remain remarkably consistent, resulting in a rather sedentary life-style.

The variation of sex and breeding status within a group allowed the evaluation of the possible effects of these factors on infestation with ecto- and endoparasites. In addition, sampling throughout the year enabled us to evaluate potential seasonal effects that may be linked to climate factors as well as increases in mobility. The variation in group size found in this species further allowed us to examine possible relationships between group size and parasite loads. Since parasites have not previously been described for the study species we firstly aimed to provide an inventory of the parasites of highveld mole-rats. In addition, we predicted that males would have higher parasite loads than females and that breeders would be more heavily parasitised than non-breeders. We further hypothesized that peaks in parasite loads would be linked to energetic bottlenecks (i.e. breeding season, winter) and thus seasonal patterns would be apparent. Lastly, we predicted that ectoparasite loads would correlate negatively with group size while this would not be the case for endoparasites.

## Results

### Ectoparasite assessment

**Figure 1:**
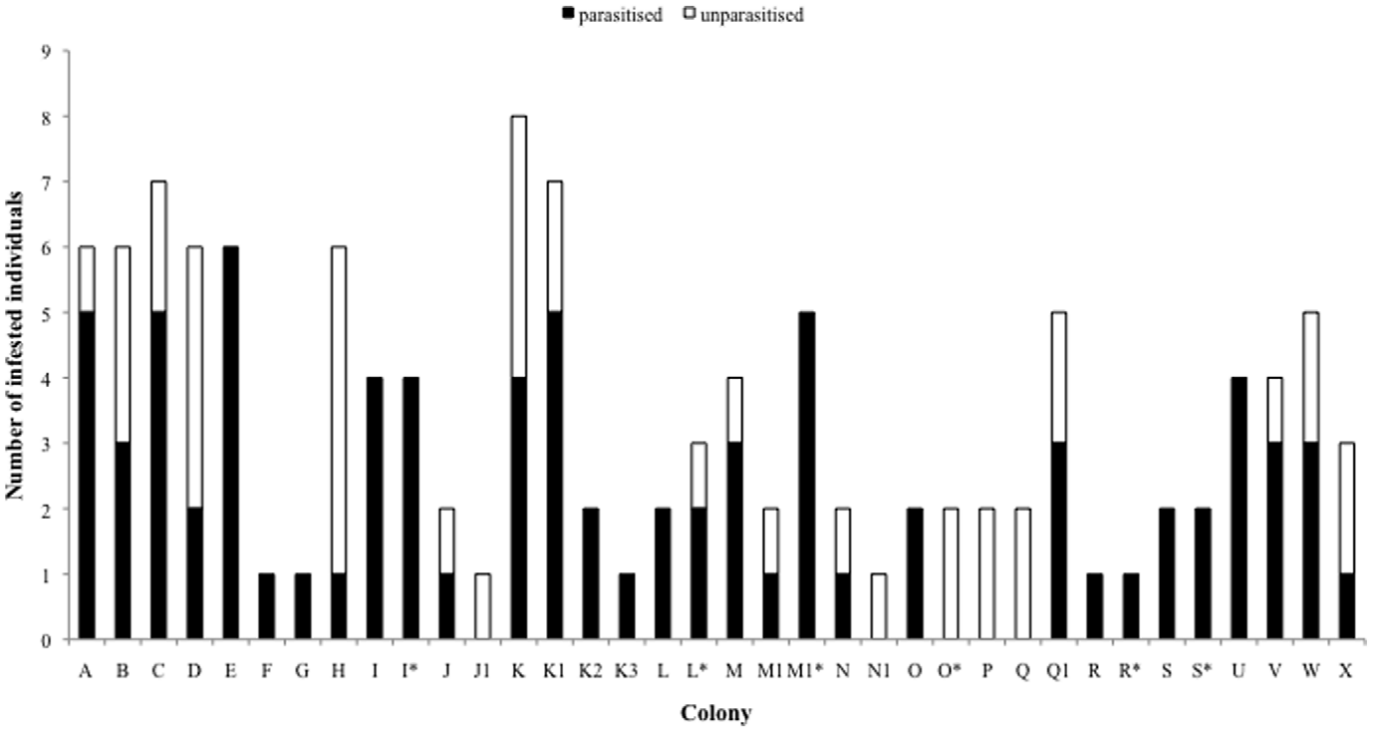
Prevalence of Laelapid mites found on highveld mole-rats during A) by colony sampled (*indicate colonies that have been sampled repeatedly) and B) during winter and summer.

A total of 88 individuals (11 BFs, 9BMs, 44NBFs, 24 NBMs) were assessed for ectoparasite loads between one and five times during this study (Table 1). Six of these animals harboured nine lice (*Linognathus* sp.) in total. The majority of ectoparasites found (99.2%), however, were gamasid mites of the genus *Androlaelaps*. They comprised three species namely *Androlaelaps scapularis*, *A. capensis* and *A. marshalli* with *A. scapularis* by far the most prevalent species. Individuals that were infested with *A. capensis* (n = 6) and *A. marshalli* (n = 6) were co-infections with *A. scapularis* with one individual harbouring all three species. Since all mites were members of the same genus, data for mites were combined for analyses. Among all animals captured mite prevalence was 65.3% and did not vary significantly with colony size (Wald χ^2^ = 0.65, df = 1, p = 0.42, Figure 1). Neither reproductive status nor sex or body mass significantly affected the prevalence of *Androlaelaps* sp. (Table 2). However, mite prevalence was significantly higher in summer (80.9%, n = 47) compared to winter (55.8%, n = 77) (Wald χ^2^ = 7.236, df = 1, p = 0.007).

**Table 1:** Summary of the parasites found and their infection parameters in highveld mole-rats.

Parasite	Total no. of parasites	Prevalence [%]	Abundance [Mean]
*Linognathus* sp.	9	7.1	0.07
*Androlaelaps scapularis*	1013	65.3	8.86
*Androlaelaps capensis*	16	7.1	0.11
*Androlaelaps marshalli*	25	7.1	0.17
*Protospirura* sp.	3	4.4	0.08
*Heligmonina* sp.	2	4.4	0.06
*Mathevotaenia* sp.	370	71.9	8.04

**Table 2:** Results of GEE's evaluating the effects of life-history traits, season and colony size on mite loads in highveld mole-rats (n = 124).

	Mite prevalence			Mite abundance		
Variable	χ2	df	p	χ2	df	p
Season	7.236	1	0.007*	10.554	1	0.001*
Reproductive status	1.599	1	0.206	0.012	1	0.912
Sex	1.480	1	0.224	0.008	1	0.927
Body mass	0.426	1	0.514	1.319	1	0.251
Colony size	0.654	1	0.419	10.082	1	0.001*

Individual and colony were included as repeated measures in the model.

*indicate significant variables.

**Figure 2:**
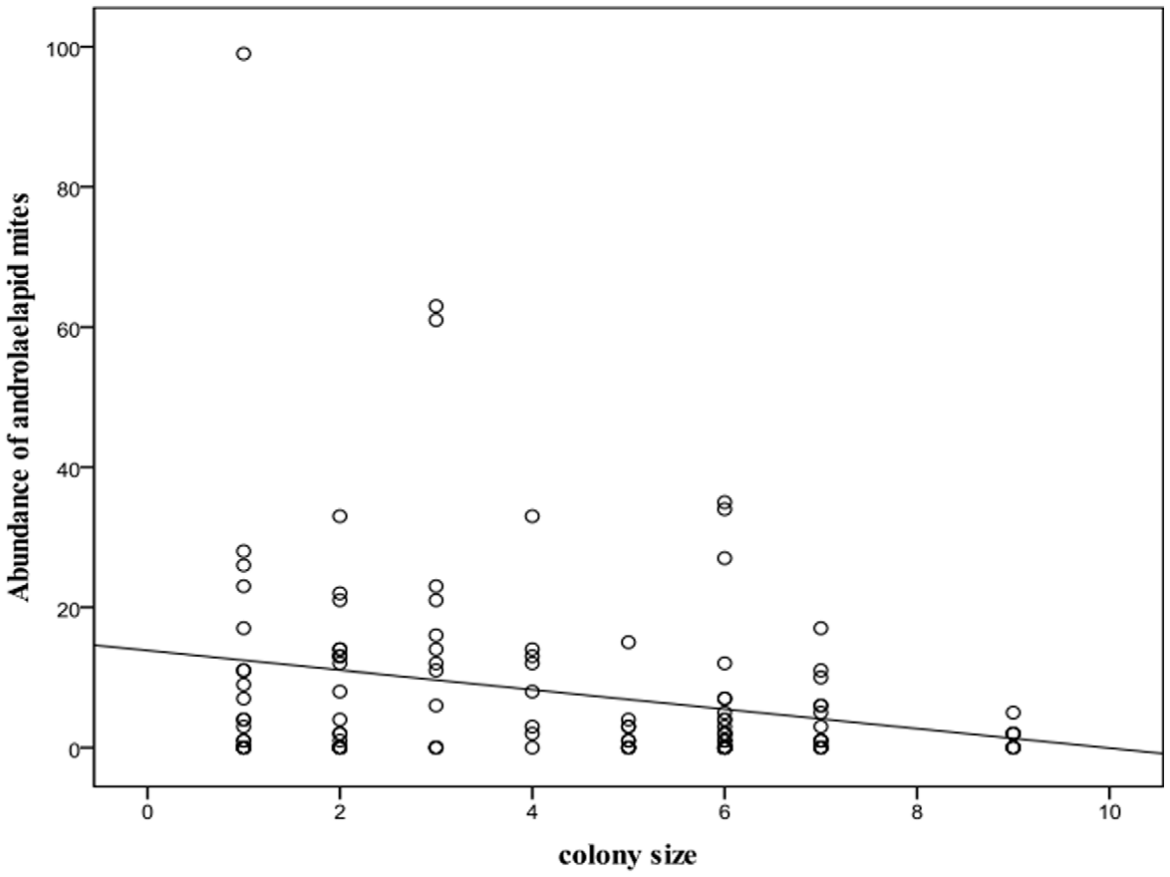
Correlation between colony size and abundance of laelapid mites found on highveld mole-rats.

The abundance of mites decreased significantly with colony size (Wald χ^2^ = 10.86, df = 1,p = 0.001, Figure 2). In contrast, reproductive status, sex and body mass had no significant effect on the abundance of mites (Table 2). Mite abundance was, however, significantly higher in summer (15.82±25.80) compared to winter (5.32±12.47) (χ^2^ = 10.55, df = 1, p = 0.001). Colony identity had a significant effect on the mite abundance (χ^2^ = 8260.75, df = 19, p<0.0001) suggesting that mite loads were more similar between colony members than between individuals from different colonies.

### Gastrointestinal parasites

From the dissections, three gastrointestinal parasites were identified (Table 1). Two genera of nematodes were found in the stomach of four animals sampled, namely *Protospirura* sp. and *Heligmonina* sp.. The majority of individuals (60.9%), however, harboured the cestode *Mathevotaenia* sp. (Anoplocephalata) in their small intestines.

**Figure 3:**
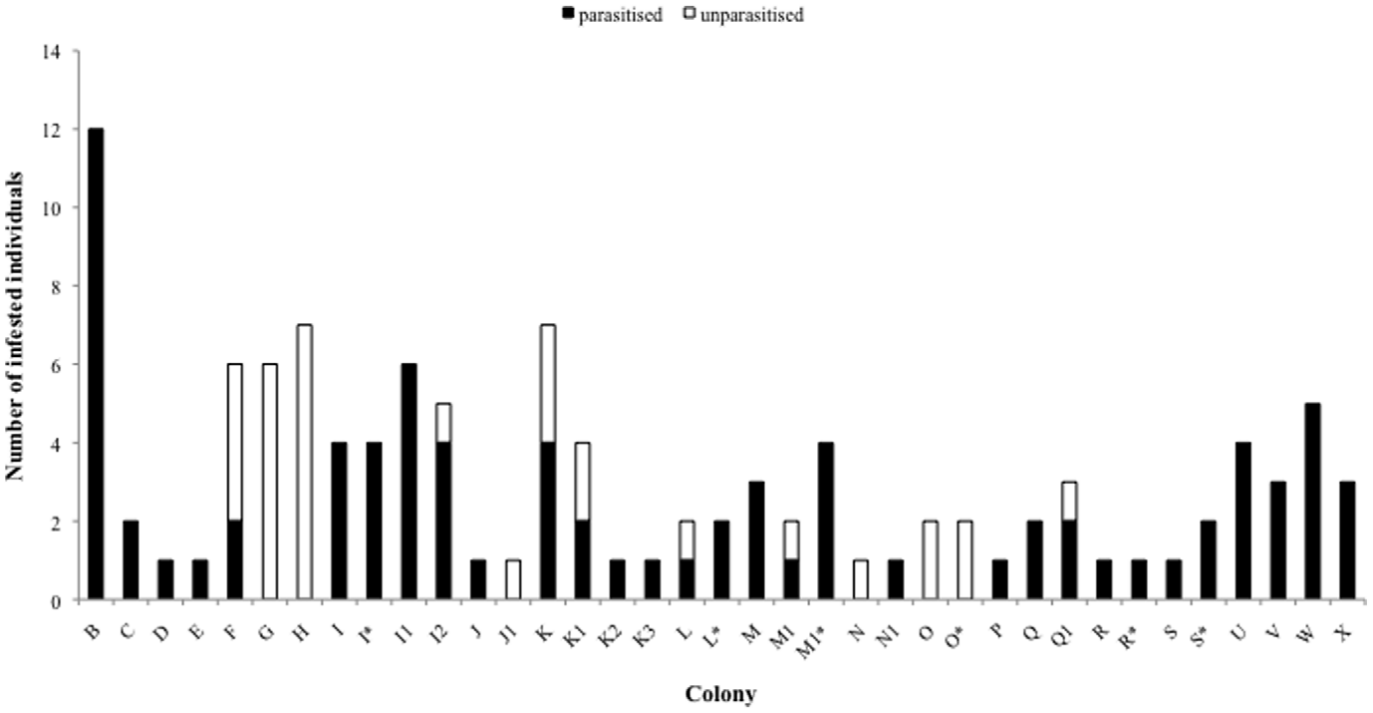
Prevalence of *Mathevotaenia* sp. in highveld mole-rats A) by colony sampled (*indicate colonies that have been sampled repeatedly) and B) during winter and summer.

**Figure 4:**
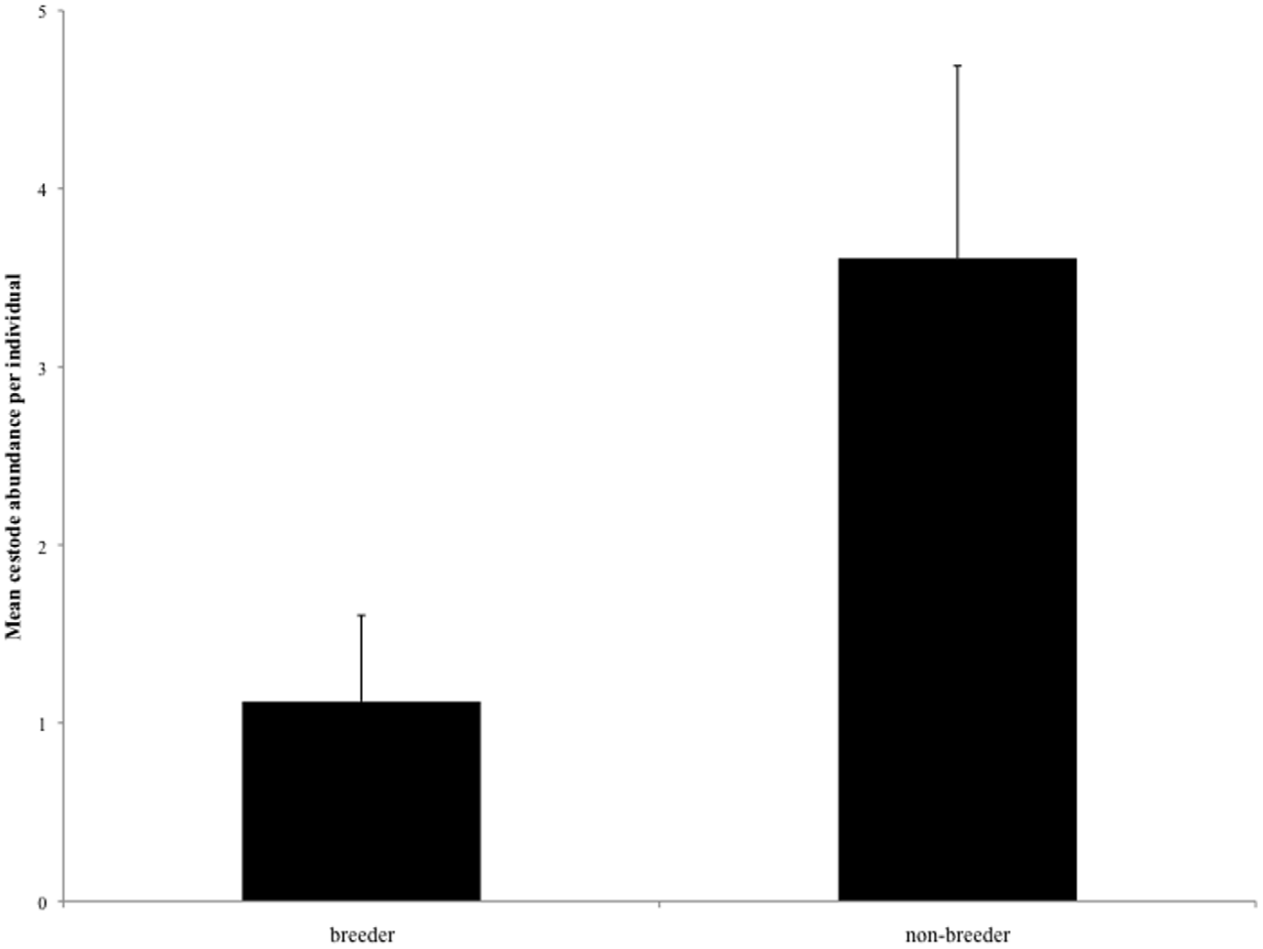
Status-dependent differences in *Mathevotaenia* sp. abundance (mean ± SD) in highveld mole-rats.

A total of 87 animals (10 BFs, 10 BMs, 50 NBFs, 17 NBMs) were assessed for prevalence of cestodes between one and five times during this study. The prevalence of cestodes was significantly higher in winter (80.2%, n = 86) than in summer (46.7%, n = 45) (χ^2^ = 10.81, df = 1, p = 0.001) (Table 2). There was no significant effect of colony size, reproductive status or sex on cestode prevalence (Table 3, Figure 3). However, parasitised individuals were significantly heavier than unparasitised ones (χ^2^ = 4.37, df = 1, p = 0.037, 95.27±20.59 g vs. 86.41±20.91 g). Cestode abundance was significantly higher in winter (12.15±4.89, *n* = 27) compared to summer (1.61±2.75, *n* = 19) (χ^2^ = 44.28, df = 1, *P*<0.0001) (Table 3). No significant effect of sex or body mass on the abundance of *Mathevotaenia* sp. was found (Table 3). However, breeders had significantly lower cestode abundances compared to non-breeders (χ^2^ = 4.98, df = 1, p = 0.026, Figure 4). Colony identity was a significant predictor of cestode intensity (χ^2^ = 36.72, df = 8, *P* = 0.0001) suggesting that the number of cestodes is more similar between colony members than between individuals originating from different colonies.

**Table 3:** Results of GEE's evaluating the effects of life-history traits, season and colony size on cestode loads in highveld mole-rats (n = 87 for prevalence, n = 46 for abundance).

	Cestode prevalence			Cestode abundance		
Variable	χ2	df	p	χ2	df	p
Season	10.81	1	0.001*	44.28	1	<0.0001*
Reproductive status	0.73	1	0.394	4.98	1	0.026*
Sex	1.68	1	0.195	1.36	1	0.243
Body mass	4.37	1	0.037*	2.31	1	0.091
Colony size	0.00	1	0.987	-	-	-

Individual and colony were included as repeated measures in the model.

*indicate significant variables.

## Discussion

The parasite fauna found in highveld mole-rats was limited to a relatively small number of parasite species and dominated by one mite and one cestode species. Similarly poor parasite faunas have been reported for a number of other subterranean rodents e.g. [51], [52], [53] and may be linked to the limited parasite exposure in the subterranean niche [54]. The sedentary subterranean life-style may also contribute to the largely lacking sex and status-specific differences in parasite loads observed in the current study. Although the study species exhibits a sex- and status-dependent body mass dimorphism and non-breeders of both sexes show a down-regulation of their reproductive axis [44] parasite loads were comparable for these groups. This could be linked to the shared habitat of group members of different sex and reproductive status [54], [55]. Locomotion and dispersal in the subterranean habitat are energetically expensive compared to above-ground locomotion [46] and accordingly dispersal events are often restricted to rainfall periods when digging becomes less costly [47]. Thus, members of a colony share the same burrow system for extended periods of time and this may result in similar parasite exposure for all colony members irrespective of their sex or breeding status. This hypothesis is further supported by the significant effect of colony identity on parasite burdens. However, significant differences in cestode abundance were apparent in highveld mole-rats and contrary to our prediction these were higher for non-breeding compared to breeding individuals. Several hypotheses may explain this finding. Firstly, breeders are likely to be older animals and thus the lower abundance in these individuals may be an indicator of an acquired immunity against cestode infestation with age as reported for other rodent species [22], [23]. A similar mechanism may also account for the negative correlation between body mass and cestode prevalence since breeders of both sexes are the heaviest animals in the colony in the study species. Alternatively, breeding animals with high *Mathevotaenia* sp. abundances could have higher mortality rates due to the dual energetic challenge and were thus underrepresented in our sample [4], [5].

We found a significant seasonal variation in both ecto- and endoparasite load, however, while both mite prevalence and abundance were higher in summer the opposite was the case for infestation with cestodes. This pattern contradicts a simple trade-off between energetic bottlenecks (e.g. reproduction, thermoregulation) and parasite defence but could be linked to the different life-history traits and transmission modes of the parasites. Mites are directly transmitted among hosts and it has been shown for other mite species that they synchronise their reproduction with that of their hosts [13], [24]. Thus, the higher mite prevalence and abundance observed during the reproductive period of the highveld mole-rat in the current study may be explained by the onset of reproduction in *Androlaelaps* sp. as well as the better availability of receptive hosts (i.e. pregnant females and juveniles) [13], [24]. However, in our study increases in mite infection were not restricted to breeding females or smaller animals suggesting that higher loads in summer are not simply a result of increased susceptibility in certain host groups. Alternatively, the increased digging activity during the wet summer season [47] together with a concurrent decrease in the time that can be devoted to grooming may result in increased exposure and ultimately mite burdens. However, the simplest explanation for the observed increases of mite loads during the wet summer season may be the positive effects of increased humidity on mites as has been shown repeatedly for various mite species e.g. [28], [30].

In contrast to mites, cestodes usually depend on an intermediate host (e.g. arthropods) for transmission. Accordingly, direct contacts between hosts alone are unlikely to result in higher cestode burdens. However, the highest cestode burdens were observed during winter when intra-colony contacts may be at peak due to thermoregulatory huddling and ecological constraints on burrowing activity as a result of low rainfall during winter [47]. This could result in higher exposure rates to helminths during this relatively sedentary period of the year as has been suggested for other subterranean rodents [54]. At the same time highveld mole-rats may be more susceptible to infestation with *Mathevotaenia* sp. due to the higher energetic constraints experienced in winter when increasing thermoregulatory demands coincide with reduced food availability [47]. Endoparasites such as *Mathevotaenia* sp. reside in the small intestines and compete directly with their hosts for incoming nutrients [25]. During periods of food shortages this could further reduce the energy available to mole-rats for parasite defence and it is likely that this further contributed to the seasonal pattern of infestation observed for this parasite.

We did not find a positive correlation between parasite loads and group sizes for either parasite species. Instead the abundance of mites decreased significantly with increasing group size while no significant pattern was observed for either prevalence or abundance of *Mathevotaenia* sp. in the study population. This corresponds closely with the findings of [39] for parasite patterns in rodents. As suggested by these authors the negative correlation between *Androlaelaps* sp. abundance and group size is likely to be a result of increased grooming rates in larger groups. The situation is more complex for the cestode in this study since the complete cycle for this parasite is unknown and thus the role of the intermediate (arthropod) host cannot be evaluated.

Authors suggesting a positive relationship between parasite loads and group size e.g. [2], [32], [33] often ignore the role intermediate hosts might play and the significance of social mechanisms as well as their effect on parasite transmission e.g. [39], [56], [57]. The biology of the former is likely to be a key factor determining burdens in the final host irrespective of group size. In addition, Wilson et al. [38] suggested that social boundaries may pose strong constraints on parasite transmission within a host population. Accordingly we found that group identity was a good predictor of parasite loads for both ecto- and endoparasites. Inter-colony contacts rates are strongly limited among mole-rats due to their subterranean life-style. Thus, while group members may experience similar exposures to parasites, dispersal constraints should result in more heterogeneous parasite communities between individuals from different groups than among group members similar to what [56] suggested for primates. This could be related to group members exploiting the same patch as parasite abundance may vary between patches. In addition, in social mole-rats, groups are largely composed of family members [58], [59]. This suggests that a common genetic make-up may render some groups less susceptible to certain parasites than others.

In summary, in highveld mole-rats sex and reproductive effort appear to contribute little to heterogeneities in parasite loads, possibly as a result of the shared burrow system exploited by all group members. Both infestation with mites and cestodes showed significant seasonal variation resulting in peaks in parasite load during summer for ectoparasites while the opposite was true for endoparasites. These differences may be related to the different transmission modes, effects of environmental factors (e.g. humidity) on the parasites as well as seasonal variation in foraging and dispersal rates of the host. Contrary to popular hypotheses larger group sizes were not associated with higher parasite loads but parasite loads varied significantly with group identity. This suggests that social boundaries may pose strong constraints on parasite transmission while within groups individuals may benefit from social behaviours such as grooming. Furthermore, different groups could exploit areas that differ in their parasite abundance or vary in their susceptibility to such parasites as a result of the kin structure of groups in the study species. It has been repeatedly shown for social hymenoptera that multiple matings increase the genetic diversity and consequently disease/parasite resistance of a group e.g. [60], [61]. However, currently nothing is known about differences in susceptibility between groups of social vertebrates.

## Materials and Methods

### Capture and housing

Mole-rats were captured from May 2008 until August 2009 in the Tshwane region (S25°46′35.45″, E28°21′37.34″) of South Africa using Hickman live-traps baited with sweet potato. Captures were initially conducted monthly (May–December 2008) but later on a bimonthly basis due to logistic constraints. To ensure that all colony members were captured, traps were only removed after three consecutive days had passed without further signs of activity at a trap site. Colonies were housed in plastic crates (49.5×28 cm) with wood shavings and paper towelling for nesting material and fed an *ad libitum* diet of fresh sweet potato and apple on a daily basis until assessed for parasites. One capture site (National Botanical Garden, Pretoria) was part of a long-term, mark-recapture study and animals were released after assessment of parasite burdens. As a result, data on endoparasite abundances were not available for these individuals (see below). All animals from this site were marked individually with a subcutaneously implanted TX1400L microchip (Identipet, Johannesburg, South Africa). Prior to release at their capture site, animals were weighed, sexed and parasite loads were assessed. Reproductive males were identified as the heaviest in the colony while non-reproductive males were markedly lighter [62]. Reproductive females were readily identifiable by the presence of elongated teats and a perforate vagina which non-reproductive females lacked [44]. Individuals below a body mass of 40 g were regarded as juveniles and excluded from analyses. A total of 23 colonies were sampled with an average group size of 3.7±2.8 individuals (range: 1–12).

### Ectoparasite assessment

All members of a colony were examined for parasites within a maximum of ten days after capture. In accordance with [63] we recorded the prevalence of parasites as the percentage of individuals that were infested and abundance as the number of parasites per host considering both infested and uninfested hosts. For the assessment of ectoparasite loads we employed a modification of the body washing methods [64]. Mole-rats were anaesthetised by placing them in a jar containing a halothane-soaked cotton wool until they were unconscious. The animals were then removed from the jar and washed in a bath of tepid soapy water while preventing water from getting into their respiratory tracts. To avoid any bias this procedure was standardised to 20 dips. The soapy water was filtered through a No. 25 U.S. Standard Sieve (710 micron screen) for ectoparasites and these were then collected and placed in 70% ethanol for preservation. Recovered specimens were cleared in lactic acid and then mounted on slides with a small amount of Hoyer's medium and placed in an oven at 37°C for approximately three days to dry. Parasites were identified and counted under a light microscope (Zeiss Axioskop, magnification 100×).

### Gastrointestinal parasites

Faecal samples were collected from all individuals sampled for endoparasites and infestation with cestodes was determined by identifying proglottids in the faecal pellets. This measure of cestode prevalence was then compared with the actual infestation for individuals that were dissected (see below) to evaluate the accuracy of this method for determining cestode prevalence. Results showed that this method had a reliability of 96% with 44 of 46 being classified correctly on the basis of faecal sampling alone. Thus, we deemed the method of sufficient accuracy to include prevalence data on the basis of faecal sampling alone from the animals that were not dissected.

For the assessment of endoparasite abundance mole-rats were euthanized with an overdose of halothane and the alimentary tract was removed. Contents of the stomach, small intestine, caecum and large intestines were examined separately under a dissection microscope (40× magnification) for the presence of gastrointestinal parasites. The helminths retrieved were counted and identified at The Royal Veterinary College (Herts), London, UK and by Dr. Kerstin Junker at the Onderstepoort Veterinary Institute, Onderstepoort, South Africa.

### Statistical analysis

The majority of ectoparasites were androlaelapid mites while the cestode *Mathevotaenia* sp. accounted for the vast majority of endoparasites (see Results section). The rarity of other parasites precluded a meaningful statistical analysis for these species and hence, only descriptive statistics are reported for rare parasite species. For the remaining parasites we employed general estimating equations (GEE) to analyse the data [65]. This method is widely used in epidemiological studies to analyse longitudinal and other correlational data particularly if they are of binary or count structure [66]. For prevalence data we specified a binomial distribution and a logit-link while a negative-binomial distribution was defined for abundance data. Individual and colony were specified as repeated measures. We added the factors sex, breeding status and season as categorical predictors to the model. For analyses, data was divided into summer (September to February) and winter (March to August). In addition, colony size and body mass were added as independent variables to the model. Colony size was not included in the GEE for cestode abundance due to the limited variation in colony sizes for this part of the analysis (five out of seven colonies had a colony size of six). Initially, all two-way interactions were included in the model, however, since none of them yielded a significant result only main effects will be reported here. We tested the effect of colony membership on parasite loads in a separate GEE to avoid parameter overload on the model [67]. All statistical analyses were performed using SPSS (Version 17.0, Chicago, Ill.).
